# Case Report: First report of blue-green neutrophilic inclusions in Epstein-Barr virus-related hemophagocytic lymphohistiocytosis: a fatal pediatric case and literature review

**DOI:** 10.3389/fmed.2026.1865629

**Published:** 2026-07-10

**Authors:** Ni Yuan, Yi Wang, Dan Yao, Yanqiang Du

**Affiliations:** 1 Department of Endocrinology, Genetics and Metabolism of Xi'an Children's Hospital, National Children's Regional Medical Center (Northwest), Children's Hospital of Xi'an Jiaotong University, Xi'an, China; 2 Department of Pediatric Intensive Care Unit of Xi'an Children's Hospital, National Children's Regional Medical Center (Northwest), Children's Hospital of Xi'an Jiaotong University, Xi'an, China

**Keywords:** case report, green inclusions, hemophagocytic lymphohistiocytosis, infectious mononucleosis, neutrophils, pediatrics

## Abstract

**Background:**

Green neutrophilic inclusions (GNIs) are rare cytoplasmic inclusions in peripheral blood smears, historically associated with acute liver failure and high mortality. Recent evidence suggests that GNIs may also occur in other critical illnesses; however, reports in pediatric populations remain scarce. Hemophagocytic lymphohistiocytosis (HLH) is a life-threatening hyperinflammatory syndrome, but GNIs have not been previously described in patients with HLH.

**Case presentation:**

We report a 9-year-and-5-month-old female with Epstein-Barr virus-related hemophagocytic lymphohistiocytosis (EBV-HLH) in whom GNIs were identified in peripheral blood neutrophils during her clinical course. The patient presented with persistent fever, progressive cytopenias, hyperferritinemia, hemophagocytosis on bone marrow aspirate, and markedly elevated cytokines. Despite intensive supportive care, including continuous renal replacement therapy and plasma exchange, her condition deteriorated rapidly. On hospital day 6, GNIs were identified in her peripheral blood neutrophils. She died of multiorgan dysfunction on hospital day 8.

**Conclusion:**

This is the first report of GNIs in a patient with EBV-HLH. GNIs expand the clinical spectrum of EBV-HLH and may serve as an ominous prognostic marker in pediatric patients.

## 1 Introduction

Green neutrophilic inclusions (GNIs), also known as blue-green inclusions, green bodies, or death bodies (1), are bright green or blue-green cytoplasmic inclusions in neutrophils or monocytes on peripheral blood smears. This morphological finding is rare (2), and easily overlooked (3). Previous studies have shown a strong association between GNIs and acute liver failure (ALF) (4), and GNIs have been proposed as a high-risk marker for mortality in ALF patients (5). Once GNIs appear in the peripheral blood of patients with ALF, death often occurs within 2–14 days (2, 6). However, in recent years, GNIs have also been identified in critically ill patients (3, 7–11), suggesting that GNIs are not specific to ALF. Nevertheless, reports of GNIs in pediatric patients remain limited (8, 12–15). Epstein-Barr virus (EBV) infection is the leading cause of infectious mononucleosis, and secondary HLH is a life-threatening complication of severe infectious mononucleosis. To our knowledge, no previous case of GNIs has been reported in patients with acute infectious mononucleosis. Here, we present the first case of GNIs in a child with Epstein-Barr virus-related hemophagocytic lymphohistiocytosis (EBV-HLH). The clinical course and management of this patient are detailed below.

## 2 Case presentation

A 9-year-and-5-month-old female patient was admitted to our hospital on September 17, 2025, with a 3-day history of fever. Her body temperature reached approximately 38.5 °C without chills or convulsions. Fever subsided with antipyretics and physical cooling but rapidly recurred. She also had paroxysmal single cough without wheezing or dyspnea. Antibiotic treatment at a primary hospital was ineffective, and she was transferred to our institution, where she was admitted for “upper respiratory infection.” Physical examination on admission: she was alert and responsive. A palpable, soft, non-tender lymph node approximately the size of a soybean was noted in the cervical region, with no adhesion to surrounding tissues. Pharyngeal congestion and grade I tonsillar enlargement were present. Breath sounds were coarse bilaterally. Other findings were unremarkable. Laboratory examination on admission: white blood cell count (WBC) 6.76 × 10^9^/L, neutrophil (NE) count 3.87 × 10^9^/L, hemoglobin (HGB) 127 g/L, platelets (PLT) 93 × 10^9^/L, C-reactive protein (CRP) 42 mg/L (reference range 0–3), procalcitonin (PCT) 27 ng/mL (reference range 0–0.05), alanine aminotransferase (ALT) 117 U/L (reference range 7–30), aspartate aminotransferase (AST) 235 U/L (reference range 14–44), lactate dehydrogenase (LDH) 2,201 U/L (reference range 120–246). Renal function, triglyceride, creatine kinase (CK), and CK-MB were normal. EBV antibody testing: EBV-viral capsid antigen IgM and IgG were positive; EBV early antigen IgG and EBV nuclear antigen IgG were negative. EBV-DNA load was 2.61 × 10^5^ copies/mL (reference range < 5.0 × 10^2^).

After admission, the patient received ceftazidime for infection, acyclovir for antiviral therapy, and glycyrrhizin for liver protection. On hospital day 3, the patient continued to have persistent fever (body temperature fluctuating between 38 °C and 39 °C). WBC, HGB, and PLT decreased to 0.65 × 10^9^/L, 62 g/L, and 51 × 10^9^/L, respectively. Ferritin was markedly elevated at 33,511 ng/ml (reference range 4.63–204). Blood and sputum cultures were negative. HLH was suspected, and she was transferred to the pediatric intensive care unit (PICU).

Upon PICU admission, physical examination: she was alert with adequate spontaneous breathing. The liver was palpated 4 cm below the costal margin and 4 cm below the xiphoid process, with soft consistency; the spleen was palpated 4 cm below the costal margin. Other findings were unchanged from admission. Further laboratory tests revealed elevated cytokines: interleukin-6 (IL-6) 302 pg/ml (reference range 0–5.4), IL-8 1,116 pg/mL (reference range 0–20.6), IL-10 3,407 pg/mL (reference range 0–12.9), tumor necrosis factor-α (TNF-α) 80 pg/mL (reference range 0–16.5), and interferon-γ (IFN-γ) >10,000 pg/mL (reference range 0–23.1); IL-1β, IL-2, IL-4, IL-5, IL-12p70, IL-17, and IFN-α were normal. Fibrinogen (FIB) was 1.45 g/L (reference range 2–4). Bone marrow aspirate showed extensive hemophagocytosis. A natural killer (NK) cell activity assay was 13% (reference range ≥ 15.11%). Soluble IL-2 receptor levels (sCD25) was 284, 600 pg/ml (reference range < 6, 400). So she was diagnosed with EBV-related HLH (EBV-HLH). Dexamethasone was administered to control inflammation. A multi-disciplinary team consisting of infectious disease specialists, pediatric intensivists and hematology and coagulation experts evaluated the patient. Considering progressive multiple organ dysfunction, chemotherapy was not initiated. Alternating continuous renal replacement therapy (CRRT) and plasma exchange (administered on alternate days) were performed.

On hospital day 4, the patient developed respiratory distress and invasive mechanical ventilation was started. On hospital day 5, the patient developed pulmonary hemorrhage, gastrointestinal bleeding, and nasal bleeding, with progressive decline in consciousness (Glasgow Coma Scale score 3). D-dimer was 14 mg/L (reference range 0–0.55), suggesting overt disseminated intravascular coagulation (DIC). Due to the laboratory testing limitations of our hospital (the hospital's clinical laboratory does not have the corresponding detection kits and technical platforms for this biomarker), FDPs was not tested. Chest computed tomography (CT) showed bilateral pneumonia with pleural effusions, multiple patchy opacities, and consolidations (Figure 1); brain CT was normal. Due to severe thrombocytopenia, hypofibrinogenemia, prolonged coagulation indices and active multiple-site bleeding caused by DIC, we administered platelet concentrates, cryoprecipitate, fibrinogen and prothrombin complex concentrates for hemostasis and coagulation factor replacement. Low-molecular-weight heparin was not used because of critical active bleeding.

**Figure 1:**
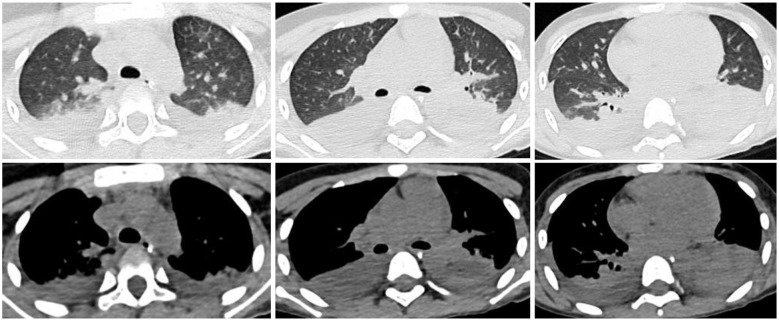
Chest computed tomography (CT) of the patient (5 days after admission). Multiple patchy opacities and consolidations were observed in both lungs. Bilateral pleural effusions with meniscus sign.

On hospital day 6, GNIs were detected exclusively in peripheral blood neutrophils (Figure 2); no inclusions were identified in monocytes or bone marrow macrophages. Figure 3 illustrates the serial laboratory trends correlating with the clinical deterioration. Specifically, following the appearance of GNIs, the patient's multiorgan dysfunction progressed rapidly despite supportive measures. Despite repeated blood product transfusions, the patient's laboratory parameters continued to deteriorate (Figure 3). On hospital day 8, the patient died of multiorgan dysfunction despite intensive supportive care.

**Figure 2:**
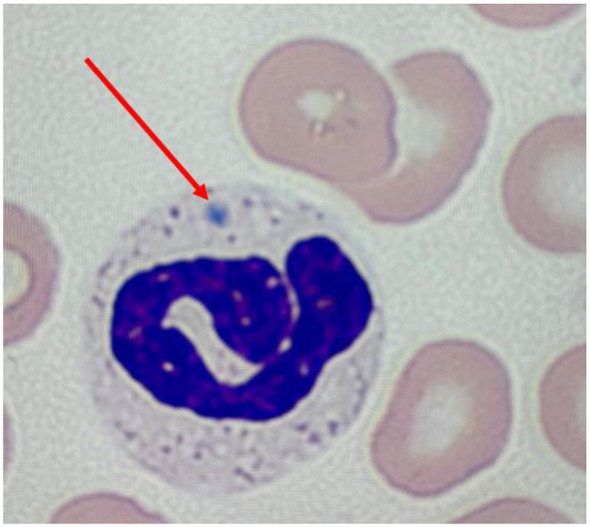
Peripheral blood smear of the patient (6 days after admission). Blue-green inclusions (arrow) in a neutrophil on peripheral blood film (Wright–Giemsa staining, × 1,000).

**Figure 3:**
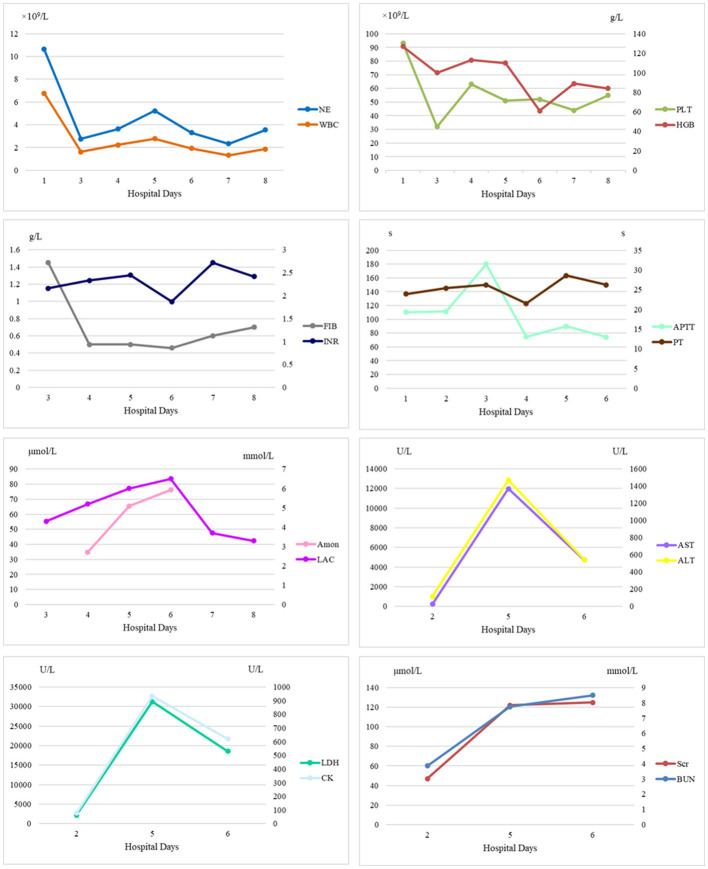
WBC and NE gradually decreased to below the normal values after admission. HGB, PLT, and FIB levels showed a declining trend despite administration of red blood cell transfusions, platelet transfusions, and fibrinogen supplementation. INR, PT, and APTT progressively increased despite replacement of coagulation substances. LAC levels progressively increased, after she was transferred to PICU. Amon, ALT, AST, LDH, CK, Scr, and BUN levels showed persistent elevations. Although these parameters exhibited a decreasing trend after initiation of blood purification, they remained above the normal ranges. WBC, white blood cell; NE, neutrophils; PLT, platelets; HGB, hemoglobin; FIB, fibrinogen; INR, international normalized ratio; APTT, activated partial thromboplastin time; PT, prothrombin time; Amon, ammonia; LAC, lactate; AST, aspartate aminotransferase; ALT, alanine aminotransferase; LDH, lactate dehydrogenase; CK, creatine kinase; BUN, blood urea nitrogen; Scr, serum creatinine; PICU, pediatric intensive care unit.

## 3 Discussion

HLH is a rare and life-threatening immune disorder characterized by uncontrolled activation of cytotoxic lymphocytes and macrophages, leading to cytokine-mediated tissue damage and multiorgan dysfunction (16). Based on the presence or absence of confirmed HLH-associated genetic abnormalities, HLH is classified into “primary HLH (pHLH)” and “secondary hemophagocytic lymphohistiocytosis (sHLH)” (17). Among these, infection-associated HLH is the most common form of sHLH, with EBV infection being the most frequently identified trigger (18). Our patient met all diagnostic criteria for sHLH and had confirmed EBV infection, leading to a definitive diagnosis of EBV-HLH (19). A limitation of our study is that the patient died shortly after admission, and thus genetic screening for pHLH could not be performed. Nevertheless, irrespective of the presence or absence of pHLH-associated genetic abnormalities, the diagnosis of EBV-HLH in this patient is unequivocal.

GNIs were first described by Smith in a 16-week-old male infant with congenital biliary atresia (20). In 2009, Harris et al. (4) first reported GNIs in peripheral blood smears of two patients with ALF. Subsequently, accumulating literature has shown that the presence of GNIs is closely associated with ALF and predicts high mortality (9, 21). Wright-Giemsa staining shows GNIs as bright blue-green granular inclusions inside neutrophils. Histochemical staining confirms GNIs are lipid-rich substances, negative for iron, PAS, bilirubin and myeloperoxidase (6, 22). Current studies indicate GNIs are mainly derived from lipofuscin released by necrotic hepatocytes (6, 22). Combined with the pathophysiology of HLH, we propose a potential mechanism for GNI formation in this case: The severe cytokine storm (especially extremely elevated IFN-γ and IL-10) in EBV-HLH causes massive hepatocyte injury and necrosis. Lipofuscin-like lipid metabolites are released into blood circulation and then phagocytosed by neutrophils, forming typical blue-green cytoplasmic inclusions. This mechanism connects HLH-specific macrophage activation and cytokine storm with GNI generation.

GNIs have been reported in patients with various viral, bacterial, fungal and protozoal infections, as well as non-infectious critical illnesses (5, 7, 9–13, 23–30), proving GNIs are not disease-specific. A previous study reported GNIs and hemophagocytosis after bee stings, but the cases did not meet HLH diagnostic criteria (10). Our patient was definitively diagnosed as EBV-HLH, so this is the first formal report of GNIs in HLH patients. Most cases with GNIs have poor prognosis, with over half of patients dying within two weeks after detection (2). Individual survivors have been reported, and changes in GNI quantity and color are correlated with clinical outcomes (23, 25). In our case, the patient died 2 days after GNI detection, consistent with the high mortality of GNI-positive critical patients.

In the future, multicenter, large-sample clinical studies are needed to confirm this phenomenon.

The search for GNIs is not technically difficult. Under Wright-Giemsa staining, GNIs can be identified by trained laboratory personnel using routine light microscopy with oil immersion. However, they are easily overlooked due to their rarity and potential confusion with Döhle bodies or toxic granulation. Currently, no guidelines mandate systematic screening for GNIs in all critically ill patients, primarily due to workload constraints and a lack of prospective evidence that early detection alters management. Nevertheless, we concur with the clinical importance of this finding. Given that EBV-HLH and other hyperinflammatory syndromes carry high mortality, we suggest that a dedicated manual smear review for GNIs be considered in patients with severe hyperinflammatory states, including HLH and acute liver failure.

## 4 Conclusion

This is the first report of GNIs in pediatric EBV-HLH. Cytokine storm related liver injury may contribute to GNIs formation. GNIs may serve as an ominous prognostic marker in EBV-HLH. Because GNIs are not technically difficult to identify, manual smear review for GNIs should be considered in severe hyperinflammatory states. Larger studies are needed to confirm these findings.

## Data Availability

The original contributions presented in the study are included in the article/supplementary material, further inquiries can be directed to the corresponding author.
